# Survey of the perceptions of key stakeholders on the attributes of the South African Notifiable Diseases Surveillance System

**DOI:** 10.1186/s12889-016-3781-7

**Published:** 2016-10-25

**Authors:** F. G. Benson, A. Musekiwa, L. Blumberg, L. C. Rispel

**Affiliations:** 1 National Department of Health, Private Bag X828, Pretoria, 0001 South Africa; 2 School of Public Health, Faculty of Health Sciences, University of the Witwatersrand, 27 St Andrews Road, Parktown, Johannesburg, 2193 South Africa; 3 Division of Global Health Protection, United States Centers for Diseases Control and Prevention (CDC), PO Box 9536, Pretoria, 0001 South Africa; 4 National Institute of Communicable Diseases, Private Bag X4, Sandringham, Johannesburg, 2131 South Africa; 5 Centre for Health Policy & DST/NRF SARChI Chair on the Health Workforce, School of Public Health, Faculty of Health Sciences, University of the Witwatersrand, 27 St Andrews Road, Parktown, Johannesburg, 2193 South Africa

**Keywords:** Notifiable diseases, Surveillance system, Acceptability, Flexibility, Simplicity, Timeliness, Usefulness

## Abstract

**Background:**

An effective and efficient notifiable diseases surveillance system (NDSS) is essential for a rapid response to disease outbreaks, and the identification of priority diseases that may cause national, regional or public health emergencies of international concern (PHEICs). Regular assessments of country-based surveillance system are needed to enable countries to respond to outbreaks before they become PHEICs. As part of a broader evaluation of the NDSS in South Africa, the aim of the study was to determine the perceptions of key stakeholders on the national NDSS attributes of acceptability, flexibility, simplicity, timeliness and usefulness.

**Methods:**

During 2015, we conducted a nationally representative cross-sectional survey of communicable diseases coordinators and surveillance officers, as well as members of NDSS committees. Individuals with less than 1 year experience of the NDSS were excluded. Consenting participants completed a self-administered questionnaire. The questionnaire elicited information on demographic information and perceptions of the NDSS attributes. Data were analysed using descriptive statistics and the unconditional logistic regression model.

**Results:**

Most stakeholders interviewed (53 %, 60/114) were involved in disease control and response. The median number of years of experience with the NDSS was 11 years (inter-quartile range (IQR): 5 to 20 years). Regarding the NDSS attributes, 25 % of the stakeholders perceived the system to be acceptable, 51 % to be flexible, 45 % to be timely, 61 % to be useful, and 74 % to be simple. Health management stakeholders perceived the system to be more useful and timely compared to the other stakeholders. Those with more years of experience were less likely to perceive the NDSS system as acceptable (OR 0.91, 95 % CI: 0.84–1.00, *p* = 0.041); those in disease detection were less likely to perceive it as timely (OR 0.10, 95 % CI: 0.01–0.96, *p* = 0.046) and those participating in National Outbreak Response Team were less likely to perceive it as useful (OR 0.38, 95 % CI: 0.16–0.93, *p* = 0.034).

**Conclusion:**

The overall poor perceptions of key stakeholder on the system attributes are a cause for concern. The study findings should inform the revitalisation and reform of the NDSS in South Africa, done in consultation and partnership with the key stakeholders.

**Electronic supplementary material:**

The online version of this article (doi:10.1186/s12889-016-3781-7) contains supplementary material, which is available to authorized users.

## Background

Notifiable disease surveillance systems (NDSS) in the 21st century should be capable of rapid identification of priority diseases that cause national, regional or public health emergencies of international concern (PHEIC). An effective and efficient NDSS could enhance the ability of a country to respond rapidly to outbreaks before they become PHEICs. Regular evaluations of the surveillance system are needed to ensure this capability, as well as relevance and usage by the key stakeholders. Furthermore, the PHEICs caused by the Ebola Virus Disease (EVD) outbreak from 2014 to 2016 [1] and the 2016 Zika virus outbreak [2] demonstrated that the status of the NDSS in each country could impact on global health security. Hence the outcome of NDSS evaluations is of relevance to the global community.

At a global level, an independent panel appointed by the World Health Organization (WHO) in response to the EVD outbreak found that countries failed to develop International Health Regulations (IHR) surveillance core capacities [3]. The panel also questioned the reliability of the annual mandatory self-administered IHR assessment questionnaires that are required by WHO of all member states [3]. These findings underscore the need for objective evaluations of the NDSS at country level. Many countries have begun to use to framework developed by the Centers for Disease Control and Prevention (CDC) to evaluate their surveillance systems. [4]

In high-income countries in Europe [5] and Australasia [6, 7], evaluations of the NDSS have focused on data quality, usefulness, acceptability, timeliness, as well as the simplicity of the system. Studies in low- and middle-income countries in the Americas [8, 9], Europe [10], Asia [11], and Africa [12–14], have found that challenges with laboratories, supervision, monitoring, organisational capacity, staffing and resources impede NDSS functioning. These findings might not be relevant to South Africa as a comparative study of the NDSS in China and the USA found that differences in context, background and resource availability among countries make it difficult to generalize findings from one country to the other [15].

**Fig. 1:**
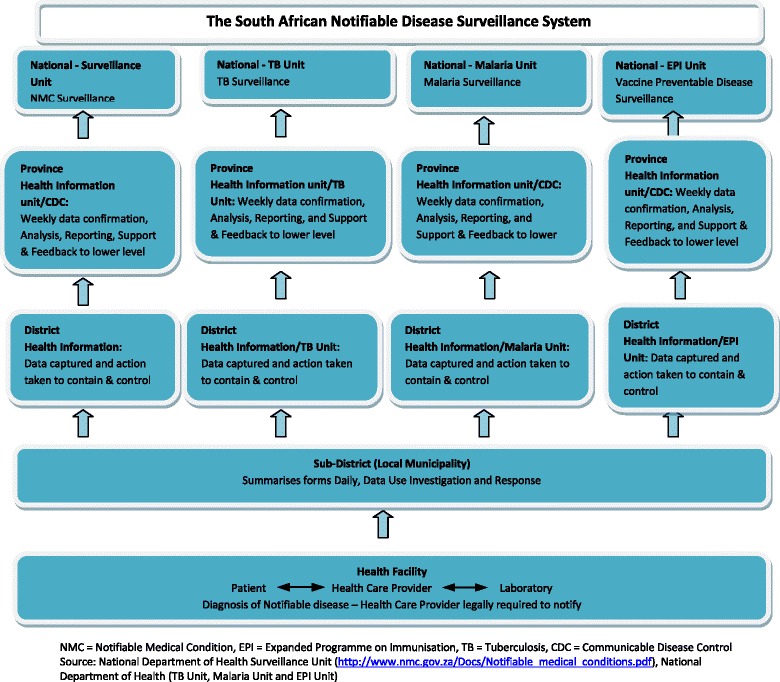
Schematic presentation of the South African Notifiable Disease Surveillance System, April 2015

The NDSS in South Africa has been in existence since the late 1970s. The NDSS in South Africa is a paper-based system that tracks 33 medical conditions. In terms of existing legislation, all health care providers are obliged to notify these 33 conditions to their local authority, which in turn reports it to the district, district to province, and province to the NDOH (Fig. 1) [16]. In the preceding 15 years, parallel surveillance systems have been developed for tuberculosis (TB), malaria and vaccine-preventable notifiable diseases. We could only find three evaluations of specific diseases at provincial level in South Africa [17–19]. However, there has been no systematic and objective evaluation of the NDSS at the national level or an evaluation of the NDSS since the adoption of the IHR in 2007. The need for such an evaluation [20] is critical, in light of health sector reforms in South Africa, which include the implementation of a National Health Insurance System [21] and the establishment of a National Public Health Institute [22]. The involvement of key NDSS stakeholders in evaluations and policy development is critical to obtain their inputs and to build mutual understanding and trust. [23–25] In this paper, the term stakeholder is used to describe individuals actively involved in the NDSS, or in structures and committees set up to deal with any aspect of the NDSS in South Africa. The stakeholders include individuals involved in disease control and response; disease detection; and health management.

As part of a broader evaluation of the NDSS in South Africa, a survey was conducted among key stakeholders in South Africa on their perceptions of the NDSS attributes of acceptability, flexibility, simplicity, timeliness and usefulness.

## Methods

During April and May 2015, we invited all communicable diseases coordinators, epidemiologists and surveillance officers at the National Department of Health (NDoH) and all nine provincial health departments, as well as members of the National Surveillance Forum, the South African Malaria Elimination Committee, the South African Expanded Programme on Immunisations Committee and the National and Provincial Outbreak Response Teams (NORT and PORT respectively), to participate in a cross-sectional survey. As experience is an important determinant of the perspectives of the stakeholders, we excluded those not working in a health related field and those with less than 1 year experience of the NDSS from the study.

### Measurement and data collection

We developed an electronic semi-structured questionnaire (Additional file 1) using the secure, web-based Research Electronic Data Capture (REDCap), programme hosted at the University of Witwatersrand [26]. In addition to socio- demographic information, the questionnaire elicited information on participants’ perceptions of the NDSS attributes of acceptability (the willingness of providers to participate in the NDSS), flexibility (adaptability to changing circumstances and needs), simplicity (ease of understanding of NDSS forms and processes), timeliness (the speed at which the provider takes the appropriate steps after an event came to her/his attention) and usefulness (whether the data contributes to outbreak response, or the prevention and control of communicable diseases or improved public health knowledge) [4].

The questionnaire consisted of one to four questions per attribute for each of the five system attributes. The questions were designed on a 7-point Likert-scale ranging from one (strongly disagree) to 7 (strongly agree). The questions were phrased in a manner that attempted to minimise an unreflective response by participants, for instance questions requiring a positive response were alternated with questions requiring negative responses so that respondents would not be tempted to continue answering all the questions using the same response. Cronbach’s alpha coefficients were calculated to determine reliability and coherence between items and ranged from 0.82 to 0.97, indicating high reliability and inter-item correlation.

We piloted the questionnaire prior to implementation, and no changes were deemed necessary. The questionnaire was in English as this is the official language used for business communication in South Africa. On 7 April 2015, we electronically sent invitations for participation in the survey to all identified key stakeholders via REDCap. The first page of the questionnaire consisted of an information sheet and we asked participants to consent before completing the survey electronically. Those not consenting were allowed to opt out. We sent four reminders to participants who did not respond after 2 weeks and we closed the survey on 31 May 2015, after 54 days from the enrolment date.

### Data analysis

We captured data entered by participants in REDCap and exported the data into STATA® 14 for cleaning and analysis. We computed frequency and summary tables to describe participants’ age, position, experience, training and roles on committees. We summarized categorical variables in tables showing frequency and percentage of each category. We also summarized numerical/measured variables using means (standard deviations) or medians (ranges) depending on whether they could be assumed to be normally distributed or skewed.

We analysed responses from 7-point Likert scale attribute questions by describing the frequency distribution for each point on the scale. In order to simplify the interpretation of the results, we then categorized the responses to each question on NDSS attributes as agree or disagree. We excluded the ‘neither agree nor disagree’ response in the analysis. We then computed the percentage of respondents who agreed with a particular attribute. We conducted a sensitivity analysis using two additional scenarios: in ‘scenario 2’, we combined the responses of participants who indicated ‘neither agree nor disagree’ with the responses of those participants who indicated ‘agree’ to the questions; in ‘scenario 3’, we combined the responses of participants who indicated ‘neither agree nor disagree’ with the responses of those participants who indicated ‘disagree’ to the questions. In the final stage of the analysis, we determined whether participants’ age, experience, training and roles with regards to the NDSS were associated with each of the attributes using the unconditional logistic regression model. The outcome variable was whether the participant agreed with the attribute or not. We calculated odds ratios (OR), 95 % confidence intervals (95 % CI) and *p*-values. *P*-values of less than 0.05 were considered to be statistically significant.

## Results

### Key stakeholders’ socio-demographic information

We enrolled a total of 141 participants and obtained a response rate of 84 %. After excluding those who did not give consent (*n* = 11), worked in a non-health related field (*n* = 9), and with less than 1 year experience with the NDSS (*n* = 7), the final sample size was 114. The median age of key stakeholders was 49 years, ranging from 26 to 69 years. The median number of years of experience in the NDSS was 11 years (inter-quartile range (IQR): 5 to 20 years). The median duration of NDSS training was 2 weeks. Most of the key stakeholders who participated in NDSS committees, participated in the NORT (43 %). (Table 1)

**Table 1:** Socio-demographic characteristics of the sample of key stakeholders^a^ of the South African Notifiable Diseases Surveillance System, April - May in 2015 (*N* = 144)

Socio- demographic Characteristics	N (%)
Age (years)	
25–34	14 (13 %)
35–44	30 (27 %)
45–54	31 (28 %)
55–64	33 (30 %)
65–69	3 (3 %)
Experience in NDSS (years)	
1–5	31 (27 %)
6–10	24 (21 %)
11–15	19 (17 %)
16–20	13 (11 %)
21–25	9 (8 %)
> 25	18 (16 %)
Training in the NDSS	70 (61 %)
Duration of Training in NDSS	
< = 1 week	31 (46 %)
2 to 4 weeks	21 (31 %)
5 to 12 weeks	7 (10 %)
13 to 26 weeks	3 (4 %)
27 to 56 weeks	5 (7 %)
57 to 104 weeks	1 (1 %)
Participation on NDSS Committees	
National Outbreak Response Team	46 (43 %)
Provincial Outbreak Response Team	26 (25 %)
Malaria Elimination Committee	21 (21 %)
Surveillance Forum	22 (22 %)
EPI Committee	19 (19 %)
Area of Responsibility	
Health Management	10 (9 %)
Disease Detection	38 (33 %)
Disease Control and Response	60 (53 %)
Other	6 (5 %)

*NDSS* notifiable disease surveillance system

^a^Not all participants answered all questions, and some participated in more than one committee

Key stakeholders’ areas of work responsibilities were regrouped into disease control and response (communicable disease co-ordinators and public health officials); disease detection (epidemiologists, surveillance officers and pathologists); health management (general health managers); and others (undetermined responsibility in the NDSS). Most key stakeholders were involved in disease control and response (53 %), and these stakeholders were younger, with a median age of 41 years, compared to 55 years in the health management group.

### Perceptions on the NDSS attributes

**Fig. 2:**
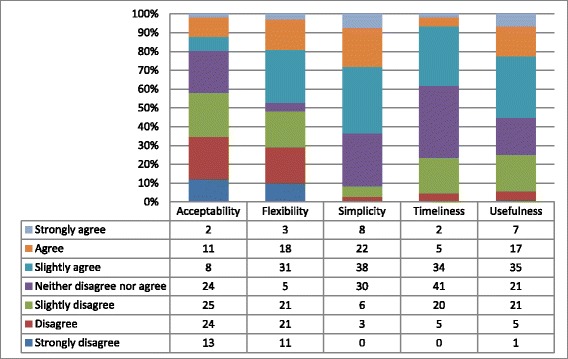
Perceptions of a sample of South African Notifiable Diseases Surveillance System stakeholders on the system attributes, April 2015

The proportion of participants who strongly agreed with any of the attributes was small, with the highest value of 7.5 % for simplicity. The proportion of participants who agreed was slightly higher at around 10 % except for timeliness. An even higher proportion slightly agreed, at around 30 %, with the exception of acceptability. The proportion of participants who neither agreed nor disagreed was also around 30 % for all attributes with the exception of flexibility. The proportion of those who slightly disagree were higher in all attributes with the exception of simplicity. The proportion of those who either disagreed or strongly disagreed was higher for acceptability and flexibility, but negligible for the other attributes. The perceptions of participants for each attribute are shown in Fig. 2.

After categorizing the responses into agree/disagree and excluding those that neither agreed or disagreed, we found that 25 % of the stakeholders perceived the NDSS to be acceptable, 51 % to be flexible, 74 % to be simple, 45 % to be timely,, and 61 % to be useful. A higher percentage of participants in Health Management perceived the system to be simple, useful and timely. Participants from the ‘Other’ category that includes those with an undetermined responsibility in the NDSS, perceived the system to be more flexible. Similarly, stakeholders participating in PORT perceived the system to be more simple and useful, compared to those in other NDSS committees. (Table 2)

**Table 2:** Key stakeholders’ perceptions on the attributes of the South African Notifiable Diseases Surveillance System by role, training and participation on committees in 2015, (%)

	Acceptability	Flexibility	Simplicity	Timeliness	Usefulness
Overall sample (*N* = 107^a^)	25	51	74	45	61
Area of Responsibility					
Health Management	14	50	100	86	100
Disease Detection	30	44	75	38	56
Disease Control and Response	27	50	72	46	58
Other	0	100	50	0	60
Training in the NDSS	29	61	77	41	68
Participation on NDSS Committees					
National Outbreak Response Team	29	49	70	45	50
Provincial Outbreak Response Team	23	53	100	52	79
Malaria Elimination Committee	13	64	71	33	67
Surveillance Forum	30	50	74	47	57
EPI Committee	31	57	73	35	61

*NDSS* notifiable disease surveillance system

*EPI* expanded programme on immunisations

^a^ = 7 participants did not record their perceptions on the NDSS

The results of the sensitivity analysis showed that the scores on simplicity and timeliness were higher in Scenario 2, when “neither agreed nor disagreed” were added to “agreed”. (Table 3)

**Table 3:** Sensitivity Analysis of Key stakeholders’ perceptions on the attributes of the South African Notifiable Diseases Surveillance System in 2015, (%) *N* = 107

	Acceptability	Flexibility	Simplicity	Timeliness	Usefulness
Scenario 1: used in the study – “Neither agree nor disagree” excluded	25	51	74	45	61
Scenario 2 : “Neither agreed nor disagreed” included as part of “agree”	42	65	92	77	75
Scenario 3 : “Neither agree nor disagree” included as “disagree”	20	36	64	38	55

Scenario 1 was used to determine the factors influencing key stakeholders’ perceptions on specific attributes of the NDSS. The logistic regression analysis revealed that the stakeholders with more years of experience were significantly less likely to perceive the NDSS system as acceptable (OR 0.91, 95 % CI: 0.84–1.00, *p* = 0.041). Participants working in disease detection were less likely to perceive the NDSS as timely (OR 0.10, 95 % CI: 0.01–0.96, *p* = 0.046), while those participating in NORT were less likely to perceive the NDSS as useful (OR 0.38, 95 % CI: 0.16–0.93, *p* = 0.034). However, there was no association between years of experience or respondents’ place of employment and the stakeholder perceptions on the NDSS attributes of flexibility and simplicity. (Table 4)

**Table 4:** Factors associated with the attributes of acceptability, simplicity, usefulness, flexibility and timeliness among key stakeholders of the South African National Diseases Surveillance System in 2015

System Attribute	Factor	Logistic Regression Analysis			Multivariate Analysis		
		Odds Ratio	95 % CI	*p*-value	Odds Ratio	95 % C I	*p*-value
Acceptability	Years of Experience	0.91	0.84–1.00	0.041*	0.91	0.84–1.00	0.041*
	Participation in NORT	1.21	0.44–3.32	0.708			
	Participation in PORT	0.76	0.24–2.44	0.651			
	Training in the NDSS	1.36	0.43–4.31	0.601			
Flexibility	Training in the NDSS	2.18	0.80–5.95	0.129			
Simplicity	Training in the NDSS	1.85	0.67–5.09	0.234			
	Participation in NORT	0.75	0.29–1.95	0.550			
Timeliness	Participation in NORT	1.17	0.50–2.76	0.714			
	Disease Detection	0.10	0.01–0.96	0.046*	0.10	0.01–0.96	0.046*
Usefulness	Training in the NDSS	2.47	1.00–6.07	0.049*	1.98	0.75–5.21	0.168
	Participation in NORT	0.41	0.17–0.98	0.045*	0.38	0.16–0.93	0.034*
	Participation in PORT	2.5	0.83–7.56	0.105			

*95 % CI* 95 % Confidence Interval

*PORT* Provincial Outbreak Response Team

*NORT* National Outbreak Response Team

*NDSS* Notifiable Diseases Surveillance System

*p values significant at 5 % level

In Scenario 2, participants younger than 35 years were more likely to perceive the NDSS as acceptable (OR 0.96, 95 % CI 0.92–0.99, *p*-value 0.044) and those trained in the NDSS perceived the system to be simpler (OR 4.74, 95 % CI 1.17–19.33 *p*-value 0.030).

## Discussion

Our findings indicate that 25 % of key stakeholders perceived the NDSS to be acceptable, 51 % to be flexible, 74 % to be simple, 45 % to be timely, and 61 % to be useful. Overall, these findings contrast with the 2014 self-administered questionnaire that South Africa submitted to the WHO on the implementation of the IHR core competencies in which it scored 100 % in surveillance core capacity [27]. The variation in scores could be explained by the different methodologies used, the differences in study periods, and because the 2014/16 EVD outbreak in West Africa could have influenced the perceptions of key stakeholders in this study.

The NDSS perceptions of key stakeholders in this study differed from the experience of the successful containment of several high profile outbreaks in South Africa since 2008, which included a novel arenavirus, Lujovirus [28]; a major cholera outbreak [29, 30]; influenza pandemic [31]; a Rift Valley Fever outbreak [32]; and a measles outbreak [33]. However, the high media attention during these events could have increased the index of suspicion and sensitivity of the surveillance system, which might not be a true reflection of the South African NDSS. Laboratories, which are not obliged by current legislation to notify diseases, may also provide information during high profile outbreaks which contribute to enhanced surveillance during these periods. Nonetheless the difference in the findings of this study and the 2014 IHR report indicates the need for more objective IHR core capacity assessments.

The study found that only 25 % of the stakeholders perceived the system to be acceptable, which implies that the stakeholders may be unwilling to participate in the system. This score of less than 50 % is similar to the finding of the 2007 study in one South African province, that found that 37 % of general practitioners indicated that they complied with the NDSS (reflection of acceptability) [17]. Comparing acceptability against the German NDSS [34] score of 90 %, the South African NDSS score was significantly lower. The participation of the health care providers is essential to ensure an effective and efficient system. Hence, this attribute needs to be addressed in the reform of the South African NDSS. Only 51 % of key stakeholders perceived the system to be flexible, implying that there are problems with the adaptability of the NDSS to changing circumstances and needs. This finding is similar to that of a qualitative evaluation study on TB surveillance in one district in the Western Cape Province of South Africa that found that although the software used was adaptable, the system did not adjust according to the changing needs [18]. It should be noted that TB has an electronic surveillance system, whereas the surveillance system for all other notifiable diseases is paper-based. Although a comparison with TB surveillance should be made with caution due to these technological differences, the adaptability of the NDSS to the development of new technology appears to lag behind. This suggests the future use of an electronic NDSS system that is responsive to the needs of various stakeholders. In this study, simplicity obtained the highest score of 74 % compared to the other NDSS attributes. This finding is comparable to a study on the Australian NDSS system in 2004 [6] which rated their operations and processes as complex. The introduction of a simple electronic NDSS system in South Africa could potentially address the perceived complexities of the NDSS [35] and also increase efficiency. The timeliness score of 45 % is also lower compared to the findings of a 2010 Ugandan study that found a score of 68–73 % [12]. Although the different methodologies of the two studies may account for the differences, the lower score in our study may imply that many health care providers and public health officials do not take prompt appropriate steps when an increase in specific diseases come to their attention through the NDSS. As prompt action is essential to contain any outbreak, timeliness must be addressed in the future reforms of the South African NDSS.

The usefulness score of 61 % was also lower than the one obtained in the Australian study that found that 94 % of participants reported reading NDSS reports and 85 % reported using the data [16]. This may indicate that there are gaps in South Africa in the utilisation of the NDSS data for outbreak response, or for prevention and control of communicable diseases. This attribute must therefore be addressed in the reform of the South African NDSS.

When considering perceptions in terms of responsibility in the system, those stakeholders involved at an operational level (disease detection and response) scored the usefulness, simplicity and timeliness of the NDSS lower than those in health management. The perceptions of health management may be an overestimation because they may have regarded the NDSS evaluation as a reflection of their own performance. Hence the scores may reflect social desirability bias. In terms of participation in NDSS committees, those participating in the provincial committees, PORT, scored the simplicity and usefulness of the NDSS higher than those in national committees. As is the case with health management, social desirability bias may again have played a role here as provinces are mainly responsible for the NDSS implementation.

Results from the logistic regression analysis showed that the stakeholders in disease detection and national committees, NORT, involved with oversight of the system, as well as those with more years of experience with the system were less likely to perceive the NDSS as acceptable, timely or useful. This may represent a true reflection of the level of functioning of the system as the surveillance officers, epidemiologists, pathologists, communicable disease coordinators and public health officials are involved with the NDSS on a daily basis in an operational and monitoring capacity – the acceptability, timeliness and usefulness of the NDSS have direct application to their daily work. On the other hand, the perceptions of oversight structures may be a reflection of their distance from the operational functioning of the NDSS.

Although the result of the sensitivity analysis in Scenario 2 showed an increase in timeliness to 77 %, this would not alter our conclusion as it still fall below a level that could be regarded as satisfactory for the effective function of the NDSS. However, in Scenario 2, a score of 92 % for simplicity is very good, and implies that no intervention is needed to improve the simplicity of the NDSS.

With regard to factors found to be associated with the perceptions in Scenario 2, the finding that training was associated with simplicity imply that addressing training needs would increase the understanding of the processes and forms used in the system. The finding that participants younger than 35 years found the system more acceptable, should inform additional training and feedback that should be used to revitalise the NDSS. Training and feedback influence the value that stakeholders at the coalface attach to the system and affect their willingness to participate in it. Without the participation of stakeholders at all levels the NDSS cannot fulfil its purpose.

The main limitation of this study was that it was based on the perceptions of individuals and not on the actual records of notifications, which is the focus of another study. Perceptions are influenced by social desirability bias among stakeholders surveyed. Although every attempt was made to include all the relevant key stakeholders at national and provincial levels, some stakeholders may not have been identified. The findings of this study will be validated through further studies on the actual records of notifications. Another limitation was the dearth of national or provincial studies on the South African NDSS to compare the research findings with. The study findings suggest the need for reforms of the South African NDSS, with particular focus on the attributes of acceptability, flexibility, timeliness and usefulness. We recommend the phased introduction of an electronic system that includes the use of mobile telephone technology to address the current perceived weaknesses in the NDSS attributes. This is because the latter has a high penetration in the South African population. In 2015 there have been some encouraging developments with regard to malaria surveillance [36] that could be built upon. The 2014–2016 EVD outbreak and the current Zika virus outbreak provide a window of opportunity that should be used to strengthen the NDSS system. We further recommend additional training and feedback to all stakeholders in the system.

At a global level, the findings of this study indicate a need for objective evaluations in support of annual IHR country submissions to the WHO. We recommend that objective assessments, using the baseline data provided in this study, be conducted every three to 5 years. This should be complemented with comparative studies of notification versus laboratory surveillance to provide a comprehensive evaluation of the NDSS in South Africa.

## Conclusions

This study found that the majority of key stakeholders scored the NDSS in South Africa low on the attributes of acceptability, flexibility, and usefulness. Factors found to be associated with key stakeholders perceptions were years of experience, training in the NDSS, age less than 35 years, participation in disease detection and NORT. The overall poor perceptions of NDSS stakeholders on the system attributes are a cause for concern. The study findings should inform the revitalisation and reform of the NDSS in South Africa to address stakeholder concerns. This should be done in consultation and in partnership with the stakeholders.

## Additional file

Additional file 1: Key Informants Perception Survey Instrument. (PDF 80 kb)

## Abbreviations

95 % CI: 95 %confidence intervals
CDC: Centers for Disease Control and Prevention
EVD: Ebola Virus Disease
IHR: International Health Regulations
IQR: inter-quartile range
NDoH: National Department of Health
NDSS: Notifiable disease surveillance system
NORT: National Outbreak Response Teams
OR: odds ratios
PHEIC: public health emergency of international concern
PORT: Provincial Outbreak Response Teams
REDCap: research electronic data capture
TB: tuberculosis
WHO: World Health Organization
