# Supplementary material for: Survey of the perceptions of key stakeholders on the attributes of the South African Notifiable Diseases Surveillance System
Source: BMC Public Health. 2016 Oct 25;16:1120. doi: 10.1186/s12889-016-3781-7 (PMC5078943; doi:10.1186/s12889-016-3781-7)
Supplement: Additional file 1: — Key Informants Perception Survey Instrument. (PDF 80 kb) [file 12889_2016_3781_MOESM1_ESM.pdf]

Key Informants Perception Survey

Introduction

My name is Dr Frew Benson. I am a doctoral student in the School of Public Health at the University of the Witwatersrand in Johannesburg.

I am doing research on the Notifiable Diseases Surveillance System, which focuses on the detection, prevention and control of public health threats in South Africa.

Why are we doing the study?

The aim of this research project is to conduct an analytical study of the National Notifiable Diseases Surveillance System of South Africa in order to inform policy and health system interventions for improvements. We believe that you can help us by sharing with us your knowledge, views and experiences of the system.

What are we asking you to do?

We would like you to complete the questionnaire which will take you about 20 minutes. Your participation is voluntary. This is not a test, so there is no right or wrong answer. It is your opinions and experiences that are important.

How do I know that the information I give you will not get out to others?

The information that you give in the questionnaire will be kept confidential. No one will know that it is you that have answered the questions. The information will not be given to your employer and will not affect your work. All questionnaires will be assigned a code. The answers given will be analysed and reported as group data.

Did you get permission to do the study?

Permission to carry out this project was obtained from the University of the Witwatersrand Research Ethics Committee. We have also obtained approval from the health authorities.

Are there any benefits and risks of participation?

Participation in this study is voluntary and there will be no direct benefits to anyone who completes the survey. Similarly there will be no negative consequences for individuals who do not want to complete the questionnaire. You will not be compensated for taking part in the study. You have the right not to complete the questionnaire.

Whom do I contact if I want more information?

If you have any questions about your rights as a study participant, or questions or concerns about any aspect of the study, you may contact the University Ethics office on (011) 717 1234. If you have questions about the research, you may also contact the Principal Researcher or the Research Supervisor:

Principal Researcher: Dr Frew Benson, National Department of Health, Pretoria Phone: +27 12 395 8094 Fax: +27 12 395 8905 Email: frewbenson@gmail.com

Research Supervisor: Professor Laetitia Rispel, School of Public Health, University of the Witwatersrand, Johannesburg. Phone: +27 11 717 2543 Email: laetitia.rispel@wits.ac.za

Consent To Participate

I have read the above information and I understand the objectives of the study. I further understand that my responses will be kept anonymous and confidential and that it is up to me whether or not to complete the questionnaire. I note that even if I choose not to complete this questionnaire, I should tick No in the space below. My refusal to participate will in no way prejudice me. I agree voluntarily to complete the questionnaire and to complete the survey only once.

◯ Yes ◯ No

Section A - Demographic Information

Are you working in a health related field or do your work relate to notifiable medical conditions ?
◯ Yes ◯ No

Do you have at least one year's experience with notifiable medical conditions or communicable diseases?
◯ yes ◯ no

In what professional category do you fall? (Only mark one - that is most relevant)
◯ Communicable Disease Coordinator /Manage
◯ Surveillance Officer/Manager.
◯ Malaria Coordinator /Manager
◯ EPI Coordinator /Manager
◯ Environmental Officer/Manager
◯ Port Health Officer
◯ TB Coordinator /Manager
◯ Medical Scientist
◯ Pathologist
◯ Epidemiologist
◯ Medical Officer
◯ Other

Specify Other Professional Category
____

How many years of experience do you have in your current portfolio?
____

How many years of experience do you have working with Notifiable Diseases?
____

What is your age (in years)?
____

In what sector are you employed?
◯ Private
◯ Public
◯ Civil or Non Governmental Organsation

Did you ever receive training on Notifiable Communicable Diseases?
◯ Yes ◯ No ◯ Not certain

What was the duration of the training? (in weeks)
____

How long ago did you receive this training? (In years with decimals for months - eg 3 yrs 3 mths ago is 3.25)
____

Did you receive any formal training in Epidemiology or Surveillance?
◯ Yes ◯ No

What level of formal training did you receive?
☐ Certificate
☐ Diploma
☐ Bachelor's degree
☐ Master's degree
☐ Doctorate
☐ Other

Other Formal training
____

When did you complete your formal training (year)?
____

	Yes	No
Are you member of the Provincial Outbreak Response Team	◯	◯
Are you member of the National Outbreak Response Team	◯	◯
Are you member of the SA Malaria Elimination Committee	◯	◯
Are you member of the Surveillance Forum	◯	◯
Are you member of the EPI Committtee	◯	◯
Are you member of any Other NDSS associated Committee	◯	◯

Specify Other NDSS associated Committee ______________________

Members of provincial health departments, please state in which province you work

- ◯ Eastern Cape
- ◯ Free State
- ◯ Gauteng
- ◯ KwaZulu-Natal
- ◯ Limpopo
- ◯ Mpumalanga
- ◯ Northern Cape
- ◯ North West
- ◯ Western Cape

Members of district health departments, please state in which district you work

- ◯ City of Johannesburg
- ◯ West Rand
- ◯ eThekwini
- ◯ uMgungundlovu
- ◯ Ugu
- ◯ Capricorn
- ◯ Vhembe
- ◯ Other

Specify other district ______________________

Section B -Knowledge and Skills on the Notifiable Diseases Surveillance System (NDSS)
Below is a list of skills that you need and use as part of your participation in the NDSS. For each, please indicate what your current level of skill is to perform the task on a scale of 1-10 (with 1 being Low skills, needing more support or training) and 10 being (Very high skills, no support or training needed)

	1 Very Low Skills	2	3	4	5 Low Average Skills	6 High Average Skills	7	8	9	10 Very High Skills
I know what the purpose of the notifiable diseases surveillance is	○	○	○	○	○	○	○	○	○	○
I know which diseases should be notified immediately on clinical suspicion	○	○	○	○	○	○	○	○	○	○
I know which diseases should be notified within 24 hours of laboratory confirmation of diagnosis	○	○	○	○	○	○	○	○	○	○
I know which diseases can be notified after 24-48 hours after laboratory confirmation of diagnosis	○	○	○	○	○	○	○	○	○	○
I know what processes should be followed in notifying a disease	○	○	○	○	○	○	○	○	○	○
I am able to train other team members on the Notifiable Diseases Surveillance System	○	○	○	○	○	○	○	○	○	○

	1 Very low skills	2	3	4	5 Low Average skills	6 High Average skills	7	8	9	10 Very High Skills
I am confident in the management of meningococcal meningitis										
I am confident in the management of measles										
I am confident in the management of typhoid										
I am able to access the latest protocols and guidelines on notifiable diseases										
I know who to consult if I am uncertain on the management of any notifiable disease										
I am able to train other team members on the management of meningococcal meningitis										
I am able to train other team members on the management of measles										
I am able to train other team members on the management of typhoid										

	1 Very low skills	2	3	4	5 Low Average skills	6 High Average skills	7	8	9	10 Very High Skills
I know what steps to follow in responding to an outbreak of a notifiable disease										
I am confident on the basic elements of an epidemiological investigation										
I am able to complete an outbreak investigation report										
I am able to make appropriate recommendations to prevent outbreaks that are similar to the one I investigated										
I am able to make appropriate recommendations to control outbreaks that are similar to the one I investigated										

I am able to train other team members on the response to notifiable diseases	○	○	○	○	○	○	○	○	○	○

	1 Very low skills	2	3	4	5 Low Average skills	6 High Average skills	7	8	9	10 Very High Skills
I am knowledgeable on the prevention of notifiable diseases	○	○	○	○	○	○	○	○	○	○
I am able to educate the community on the prevention of notifiable diseases	○	○	○	○	○	○	○	○	○	○
I am able to train other team members on the prevention of communicable diseases	○	○	○	○	○	○	○	○	○	○

Section C - Perceptions on attributes of the Notifiable Diseases Surveillance System

Listed below are statements on Attributes of the Notifiable Disease Surveillance. Using the provided scale state how strongly you agree or disagree with the statement

	Strongly disagree	Disagree	Disagree slightly	Neither agree or disagree	Agree slightly	Agree	Strongly Agree
The form used to notify diseases is easy to understand	○	○	○	○	○	○	○
The form used to notify diseases takes a long time to fill in	○	○	○	○	○	○	○
The notification process is easy to comply with	○	○	○	○	○	○	○
Clinicians are not willing to participate in the notifiable disease surveillance system	○	○	○	○	○	○	○
Clinicians do not notify meningococcal meningitis within 24 hours of clinical suspicion	○	○	○	○	○	○	○
Clinicians notify measles within 24 hours of diagnosis	○	○	○	○	○	○	○
Clinicians notify typhoid within 24 hours of diagnosis	○	○	○	○	○	○	○
Outbreak response teams do not respond timeously to an outbreak	○	○	○	○	○	○	○
Data obtained through the notifiable disease surveillance system is not used for outbreak response	○	○	○	○	○	○	○
Data obtained through the notifiable disease surveillance system is used for policy and guideline formulation	○	○	○	○	○	○	○
Data obtained through the notifiable disease surveillance system do not contribute to knowledge on the prevention and control of infectious diseases	○	○	○	○	○	○	○
The notifiable disease surveillance system has been changed to meet changing circumstances and needs in the last decade	○	○	○	○	○	○	○

The department provides regular feedback to doctors or nurses on notifiable diseases	○	○	○	○	○	○	○
Lack of facility supervision does not impact on compliance with the system	○	○	○	○	○	○	○

Section D - Other Comments on the Notifiable Diseases Surveillance System

	Very Poor	Poor	Satisfactory	Good	Very Good
What in your perception is the availability of staff for the notifiable diseases surveillance system at national level?	○	○	○	○	○
Availability of staff for the NDSS at provincial level?	○	○	○	○	○
Availability of staff for the NDSS at district level?	○	○	○	○	○
Availability of staff for the NDSS at facility level?	○	○	○	○	○

	Very poor	Poor	Satisfactory	Good	Very Good
What in your perception is the level of investment of funding for the notifiable diseases surveillance system at national level?	○	○	○	○	○
Investment of funding for the NDSS at provincial level?	○	○	○	○	○
Investment of funding for the NDSS at district level?	○	○	○	○	○
Investment of funding for the NDSS at facility level?	○	○	○	○	○

	Very Poor	Poor	Satisfactory	Good	Very Good
What in your perception is the organisational capacity for the notifiable diseases surveillance system at national level?	○	○	○	○	○
The organisational capacity for the NDSS at provincial level?	○	○	○	○	○
The organisational capacity for the NDSS at district level?	○	○	○	○	○
The organisational capacity for the NDSS at facility level?	○	○	○	○	○

Please indicate to what extent the following interventions would benefit the notifiable diseases surveillance system (on a scale of 1 -10, with 1= No benefit and 10 = Maximum benefit)

	1 No Benefit	2	3	4	5	6	7	8	9	10 Maximum Benefit
Addressing staffing gaps	○	○	○	○	○	○	○	○	○	○
Addressing the gaps in the organisational capacity of the department	○	○	○	○	○	○	○	○	○	○
Investing more financial resources in the system	○	○	○	○	○	○	○	○	○	○
Introduction of the use of an electronic system	○	○	○	○	○	○	○	○	○	○
Introduction of mobile technology	○	○	○	○	○	○	○	○	○	○

Do you have any further comments on the Notifiable Diseases Surveillance System

____

Do you have any recommendations on how to improve the Notifiable Diseases Surveillance System?

____
